# (*Z*)-*N*′-[(*E*)-4-Meth­oxy­benzyl­idene]-2-(meth­oxy­imino)-2-{2-[(2-methyl­phen­oxy)meth­yl]phen­yl}acetohydrazide

**DOI:** 10.1107/S2414314620010603

**Published:** 2020-08-07

**Authors:** Chetan Shrimandhar Shripanavar, Ray J. Butcher

**Affiliations:** a Chetan S Laboratory Pvt. LTD Pattankudi-591238, Karnataka, India; b Department of Chemistry, Howard University, 525 College Street NW, Washington DC 20059, USA; University of Aberdeen, Scotland

**Keywords:** crystal structure, kresoxim-methyl derivatives, fungicide

## Abstract

The title compound, C_25_H_25_N_3_O_4_, crystallizes with two mol­ecules in the asymmetric unit. In both mol­ecules, the central ethane hydrazide moiety is almost planar. In the crystal, asymmetric, bifurcated N—H⋯(N,O) hydrogen bonds link the mol­ecules into [100] chains. The packing is consolidated by weak C—H⋯O inter­actions.

## Abstract 2

The title compound crystallizes with two mol­ecules in the asymmetric unit. In the crystal, asymmetric, bifurcated N—H⋯(N,O) hydrogen bonds link the mol­ecules into [100] chains. The packing is consolidated by weak C—H⋯O inter­actions.

## Graphical abstract

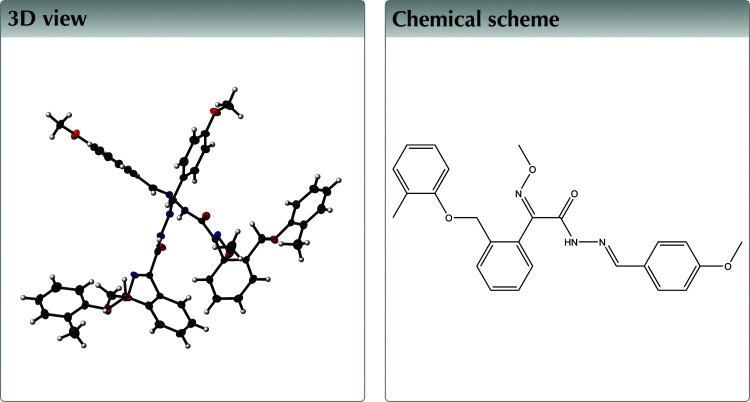

## Structure description

Kresoxim-methyl derivatives (Cao *et al.*, 2017 ▸) are broad-spectrum fungicides (*e.g.*, Grossmann & Retzlaff, 1997 ▸), have a site-specific action (Olaya *et al.*, 1998 ▸) and high efficiency (Patel *et al.*, 2012 ▸) against various diseases of agricultural crops (Balba, 2007 ▸). As these types of compounds are easily metabolized in nature as well as in living systems, their modifications are of importance in order to improve their activity (Balba, 2007 ▸). In order to increase the activity of the starting compounds (Kant *et al.* 2012 ▸), it is necessary to modify the basic skeleton and initiate a structural investigation of different derivatives of kresoxim-methyl derivatives and in that light we previously reported the crystal structure of a related derivative, (2*E*)-2-meth­oxy­imino-2-{2-[(2-methyl­phen­oxy)meth­yl]phen­yl}-*N*′-(4-nitro­benzyl­idene)ethano­hydrazide (Shripanavar & Butcher, 2015 ▸). This paper is a continuation in this series.

**Figure 1:**
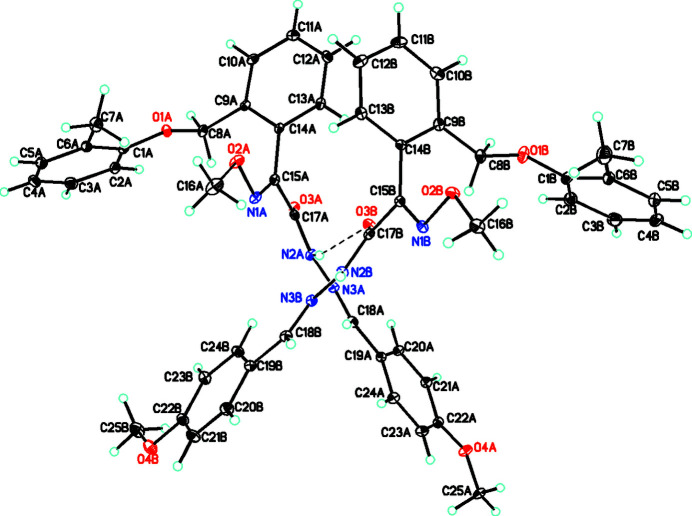
The mol­ecular structure of the two independent mol­ecules of the title compound with hydrogen bonds shown as dashed lines. Atomic displacement parameters are at the 30% probability level.

The title compound crystallizes with two mol­ecules, *A* and *B*, in the asymmetric unit, as shown in Fig. 1 ▸. Both mol­ecules exhibit similar conformations. For mol­ecule *A*, the central ethane hydrazide moiety N2*A*/N3*A*/C17*A*/O3*A* is close to planar with an r.m.s. deviation of 0.02 Å for the fitted atoms. The dihedral angles between these atoms and the adjacent rings are 17.1 (2)° (C19*A* meth­oxy­phenyl ring) and 79.9 (2)° (C14*A* benzene ring). This latter ring and the C1*A* toluyl ring are almost perpendicular, with a dihedral angle of 82.1 (1)°

For mol­ecule *B*, the central ethane hydrazide moiety N2*B*/N3*B*/C17*B*/O3*B* is also close to planar, with an r.m.s. deviation of 0.01 Å for the fitted atoms. The equivalent dihedral angles to those in mol­ecule *A* are 13.3 (2), 80.5 (2) and 86.1 (1)°, respectively.

**Figure 2:**
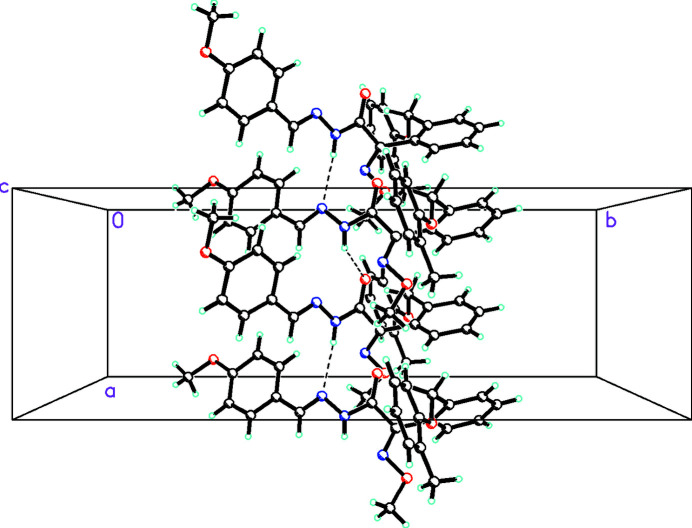
Mol­ecules of the title compound are linked by N—H⋯N and N—H⋯O inter­actions (shown as dashed lines) into a zigzag chain propagating in the *a-*axis direction.

In the crystal, bifurcated, asymmetric N—H⋯(N,O) hydrogen bonds (Table 1 ▸) link alternating *A* and *B* mol­ecules into a zigzag chain propagating in the *a*-axis direction, as shown in Fig. 2 ▸. It is of inter­est that the shorter bond is to an O atom in one of these links and to an N atom in the other. The packing is consolidated by weak C—H⋯O inter­actions.

A search for related structures resulted in two close matches. The first is the parent azide from which the current structure is derived (Chopra *et al.*, 2004 ▸) and the second is that with a nitro substituent instead of a meth­oxy substituent on the phenyl ring (CSD refcode JUFCIY; Shripanavar & Butcher, 2015 ▸). The major difference between the meth­oxy and nitro derivatives lies in the dihedral angles between the central benzene rings and the *o*-tolyl groups at the other end of the mol­ecule: in the current structure these are 82.1 (1) and 86.1 (1)° while in JUFCIY the equivalent angles are 46.98 (5) and 48.23 (4)°.

## Synthesis and crystallization

(2*E*)-2-(Meth­oxy­imino)-2-{2-[(2-methyl­phen­oxy)meth­yl]phen­yl}ethano­hydrazide (3.13 g, 0.01 mol) was refluxed for 8 h with *p*-meth­oxy­benzaldehyde (1.36 g, 0.01 mol) in 20 ml of absolute ethanol with the addition of 5 drops of glacial acetic acid to obtain a white-colored product. This was dissolved in DMSO and, by the process of slow evaporation, colourless needles of the title compound grew.

## Refinement

Crystal data, data collection and structure refinement details are summarized in Table 2 ▸.

## Supplementary Material

Crystal structure: contains datablock(s) I. DOI: 10.1107/S2414314620010603/hb4356sup1.cif

Structure factors: contains datablock(s) I. DOI: 10.1107/S2414314620010603/hb4356Isup2.hkl

Supporting information file. DOI: 10.1107/S2414314620010603/hb4356Isup3.cml

CCDC reference: 2020760

Additional supporting information:  crystallographic information; 3D view; checkCIF report

## Figures and Tables

**Table 1:** Hydrogen-bond geometry (Å, °)

*D*—H⋯*A*	*D*—H	H⋯*A*	*D*⋯*A*	*D*—H⋯*A*
N2*A*—H2*NA*⋯O3*B*	0.90 (5)	2.04 (5)	2.797 (5)	141 (4)
N2*A*—H2*NA*⋯N3*B*	0.90 (5)	2.54 (5)	3.331 (5)	147 (4)
C16*A*—H16*B*⋯O3*A* ^i^	0.98	2.51	3.353 (6)	144
C18*A*—H18*A*⋯N3*B*	0.95	2.60	3.494 (5)	158
N2*B*—H2*NB*⋯O3*A* ^i^	0.87 (5)	2.50 (5)	2.995 (5)	117 (4)
N2*B*—H2*NB*⋯N3*A* ^i^	0.87 (5)	2.29 (5)	3.155 (5)	172 (4)

Symmetry code: (i) 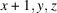 .

**Table 2:** Experimental details

Crystal data	
Chemical formula	C_25_H_25_N_3_O_4_
*M*_r_	431.48
Crystal system, space group	Orthorhombic, *P*2_1_2_1_2_1_
Temperature (K)	100
*a*, *b*, *c* (Å)	7.8041 (6), 22.8879 (16), 25.2670 (16)
*V* (Å^3^)	4513.2 (5)
*Z*	8
Radiation type	Mo *K*α
μ (mm^−1^)	0.09
Crystal size (mm)	0.55 × 0.10 × 0.08

Data collection	
Diffractometer	Bruker APEXII CCD
Absorption correction	Multi-scan (*SADABS*; Sheldrick, 1996 ▸)
*T*_min_, *T*_max_	0.370, 0.746
No. of measured, independent and observed [*I* > 2σ(*I*)] reflections	21010, 11096, 6150
*R*_int_	0.118
(sin θ/λ)_max_ (Å^−1^)	0.667

Refinement	
*R*[*F* ^2^ > 2σ(*F* ^2^)], *wR*(*F* ^2^), *S*	0.069, 0.136, 0.93
No. of reflections	11096
No. of parameters	591
H-atom treatment	H atoms treated by a mixture of independent and constrained refinement
Δρ_max_, Δρ_min_ (e Å^−3^)	0.24, −0.29

Computer programs: *APEX2* and *SAINT* (Bruker, 2005 ▸), *SHELXT* (Sheldrick 2015*a* ▸), *SHELXL2018/3* (Sheldrick, 2015*b* ▸) and *SHELXTL* (Sheldrick 2008 ▸).

## full crystallographic data

### Crystal data

**Table:**

C_25_H_25_N_3_O_4_	*D*_x_ = 1.270 Mg m^−^^3^
*M**_r_* = 431.48	Mo *K*α radiation, λ = 0.71073 Å
Orthorhombic, *P*2_1_2_1_2_1_	Cell parameters from 3813 reflections
*a* = 7.8041 (6) Å	θ = 2.7–21.7°
*b* = 22.8879 (16) Å	µ = 0.09 mm^−^^1^
*c* = 25.2670 (16) Å	*T* = 100 K
*V* = 4513.2 (5) Å^3^	Needle, colorless
*Z* = 8	0.55 × 0.10 × 0.08 mm
*F*(000) = 1824	

### Data collection

**Table:**

Bruker APEXII CCD diffractometer	6150 reflections with *I* > 2σ(*I*)
φ and ω scans	*R*_int_ = 0.118
Absorption correction: multi-scan (SADABS; Sheldrick, 1996)	θ_max_ = 28.3°, θ_min_ = 1.8°
*T*_min_ = 0.370, *T*_max_ = 0.746	*h* = −10→10
21010 measured reflections	*k* = −30→30
11096 independent reflections	*l* = 0→33

### Refinement

**Table:**

Refinement on *F*^2^	Primary atom site location: structure-invariant direct methods
Least-squares matrix: full	Secondary atom site location: difference Fourier map
*R*[*F*^2^ > 2σ(*F*^2^)] = 0.069	Hydrogen site location: mixed
*wR*(*F*^2^) = 0.136	H atoms treated by a mixture of independent and constrained refinement
*S* = 0.93	*w* = 1/[σ^2^(*F*_o_^2^) + (0.0506*P*)^2^] where *P* = (*F*_o_^2^ + 2*F*_c_^2^)/3
11096 reflections	(Δ/σ)_max_ < 0.001
591 parameters	Δρ_max_ = 0.24 e Å^−^^3^
0 restraints	Δρ_min_ = −0.28 e Å^−^^3^

### Special details

**Table:**

Geometry. All esds (except the esd in the dihedral angle between two l.s. planes) are estimated using the full covariance matrix. The cell esds are taken into account individually in the estimation of esds in distances, angles and torsion angles; correlations between esds in cell parameters are only used when they are defined by crystal symmetry. An approximate (isotropic) treatment of cell esds is used for estimating esds involving l.s. planes.
Refinement. H atoms were positioned geometrically and refined as riding: C–H = 0.95–0.98 Å with *U_iso_*(H) = 1.5*U*_eq_(C) for methyl H atoms and = 1.2*U_eq_*(C) for other H atoms. Amine H atoms were refined isotropically. The absolute structure could not be determined in the present refinement.

### Fractional atomic coordinates and isotropic or equivalent isotropic displacement parameters (Å^2^)

**Table:**

	*x*	*y*	*z*	*U*_iso_*/*U*_eq_	
O1A	0.1243 (4)	0.63579 (12)	0.58797 (10)	0.0203 (7)	
O2A	0.4283 (4)	0.59609 (12)	0.50663 (11)	0.0248 (7)	
O3A	−0.0986 (4)	0.54505 (12)	0.44415 (10)	0.0199 (7)	
O4A	−0.1398 (4)	0.23532 (12)	0.23792 (11)	0.0256 (7)	
N1A	0.3155 (5)	0.55457 (15)	0.48668 (13)	0.0206 (8)	
N2A	0.1290 (5)	0.48652 (15)	0.42402 (13)	0.0176 (8)	
H2NA	0.241 (6)	0.487 (2)	0.4155 (17)	0.032 (14)*	
N3A	0.0302 (4)	0.44522 (14)	0.39725 (13)	0.0168 (8)	
C1A	0.0846 (6)	0.61249 (17)	0.63698 (16)	0.0198 (10)	
C2A	−0.0757 (6)	0.59077 (18)	0.65070 (16)	0.0234 (11)	
H2AA	−0.165550	0.589987	0.625330	0.028*	
C3A	−0.1039 (7)	0.57024 (19)	0.70162 (17)	0.0311 (12)	
H3AA	−0.213469	0.555692	0.711348	0.037*	
C4A	0.0281 (7)	0.5710 (2)	0.73823 (18)	0.0352 (13)	
H4AA	0.009174	0.556932	0.773108	0.042*	
C5A	0.1868 (7)	0.59222 (19)	0.72408 (17)	0.0295 (12)	
H5AA	0.276160	0.592626	0.749602	0.035*	
C6A	0.2192 (6)	0.61295 (19)	0.67364 (16)	0.0226 (11)	
C7A	0.3921 (6)	0.6368 (2)	0.65789 (17)	0.0302 (11)	
H7AA	0.473484	0.631143	0.686986	0.045*	
H7AB	0.381862	0.678614	0.650086	0.045*	
H7AC	0.433331	0.616202	0.626368	0.045*	
C8A	−0.0129 (5)	0.63901 (19)	0.54977 (15)	0.0205 (10)	
H8AA	−0.111818	0.660116	0.565160	0.025*	
H8AB	−0.050807	0.599158	0.540039	0.025*	
C9A	0.0504 (5)	0.67058 (17)	0.50159 (15)	0.0162 (9)	
C10A	0.0117 (5)	0.72957 (18)	0.49412 (17)	0.0209 (10)	
H10A	−0.050877	0.750007	0.520526	0.025*	
C11A	0.0631 (6)	0.75865 (19)	0.44895 (16)	0.0230 (10)	
H11A	0.033926	0.798619	0.444264	0.028*	
C12A	0.1562 (6)	0.72999 (19)	0.41069 (16)	0.0235 (11)	
H12A	0.191614	0.750215	0.379728	0.028*	
C13A	0.1986 (5)	0.67155 (19)	0.41732 (16)	0.0200 (10)	
H13A	0.264424	0.651960	0.391140	0.024*	
C14A	0.1445 (5)	0.64151 (17)	0.46245 (15)	0.0150 (9)	
C15A	0.1822 (5)	0.57772 (18)	0.46630 (15)	0.0165 (9)	
C16A	0.5739 (6)	0.5653 (2)	0.52806 (19)	0.0363 (13)	
H16A	0.651842	0.593312	0.544900	0.054*	
H16B	0.634176	0.544977	0.499461	0.054*	
H16C	0.534777	0.536864	0.554370	0.054*	
C17A	0.0559 (5)	0.53533 (18)	0.44343 (15)	0.0170 (9)	
C18A	0.1193 (6)	0.40439 (17)	0.37641 (15)	0.0185 (10)	
H18A	0.238680	0.403264	0.383728	0.022*	
C19A	0.0478 (5)	0.35954 (17)	0.34207 (15)	0.0171 (9)	
C20A	−0.1141 (6)	0.36428 (18)	0.31907 (16)	0.0199 (10)	
H20A	−0.184670	0.396864	0.327234	0.024*	
C21A	−0.1724 (5)	0.32207 (18)	0.28458 (16)	0.0209 (10)	
H21A	−0.282790	0.325688	0.269081	0.025*	
C22A	−0.0700 (6)	0.27419 (18)	0.27242 (16)	0.0204 (10)	
C23A	0.0922 (6)	0.26902 (18)	0.29479 (16)	0.0225 (10)	
H23A	0.162775	0.236427	0.286587	0.027*	
C24A	0.1494 (6)	0.31165 (18)	0.32895 (16)	0.0216 (10)	
H24A	0.260637	0.308325	0.343902	0.026*	
C25A	−0.0391 (6)	0.18549 (18)	0.22374 (17)	0.0271 (11)	
H25A	−0.107942	0.158933	0.201989	0.041*	
H25B	−0.001838	0.165182	0.255892	0.041*	
H25C	0.061612	0.198218	0.203624	0.041*	
O1B	0.6249 (4)	0.60868 (13)	0.20834 (11)	0.0309 (8)	
O2B	0.9256 (4)	0.56148 (12)	0.28949 (11)	0.0228 (7)	
O3B	0.4078 (4)	0.52442 (12)	0.36353 (11)	0.0230 (7)	
O4B	0.2670 (4)	0.25087 (14)	0.59683 (12)	0.0317 (8)	
N1B	0.8125 (4)	0.52425 (15)	0.31583 (13)	0.0173 (8)	
N2B	0.6328 (5)	0.47020 (15)	0.39350 (13)	0.0162 (8)	
H2NB	0.743 (6)	0.466 (2)	0.3966 (17)	0.037 (16)*	
N3B	0.5289 (4)	0.43565 (15)	0.42449 (13)	0.0167 (8)	
C1B	0.5841 (6)	0.58351 (19)	0.16043 (16)	0.0262 (11)	
C2B	0.4256 (7)	0.5602 (2)	0.14837 (17)	0.0306 (12)	
H2BA	0.336734	0.560183	0.174059	0.037*	
C3B	0.3972 (8)	0.5369 (2)	0.09889 (18)	0.0402 (14)	
H3BA	0.288491	0.520856	0.090306	0.048*	
C4B	0.5270 (8)	0.5370 (2)	0.06179 (19)	0.0421 (15)	
H4BA	0.506915	0.521500	0.027483	0.050*	
C5B	0.6854 (8)	0.5596 (2)	0.07435 (18)	0.0411 (15)	
H5BA	0.773494	0.559106	0.048392	0.049*	
C6B	0.7201 (7)	0.5830 (2)	0.12396 (17)	0.0288 (12)	
C7B	0.8927 (7)	0.6065 (2)	0.13842 (19)	0.0436 (14)	
H7BA	0.972592	0.599935	0.109095	0.065*	
H7BB	0.934755	0.586470	0.170138	0.065*	
H7BC	0.883811	0.648494	0.145493	0.065*	
C8B	0.4962 (6)	0.6076 (2)	0.24845 (15)	0.0219 (10)	
H8BA	0.472415	0.566859	0.259295	0.026*	
H8BB	0.388680	0.625069	0.234935	0.026*	
C9B	0.5618 (5)	0.64225 (18)	0.29482 (16)	0.0189 (10)	
C10B	0.5292 (5)	0.70184 (19)	0.29708 (18)	0.0233 (10)	
H10B	0.467486	0.719908	0.269115	0.028*	
C11B	0.5846 (6)	0.73523 (19)	0.33896 (17)	0.0251 (11)	
H11B	0.561065	0.775948	0.339720	0.030*	
C12B	0.6743 (6)	0.7096 (2)	0.37989 (18)	0.0262 (11)	
H12B	0.712485	0.732556	0.408898	0.031*	
C13B	0.7085 (5)	0.65023 (18)	0.37850 (16)	0.0179 (10)	
H13B	0.769870	0.632444	0.406698	0.022*	
C14B	0.6535 (5)	0.61669 (17)	0.33608 (15)	0.0150 (9)	
C15B	0.6889 (5)	0.55231 (17)	0.33677 (15)	0.0146 (9)	
C16B	1.0620 (5)	0.52601 (19)	0.26879 (17)	0.0265 (11)	
H16D	1.140421	0.550498	0.248146	0.040*	
H16E	1.014032	0.495489	0.245983	0.040*	
H16F	1.124637	0.507882	0.298122	0.040*	
C17B	0.5625 (6)	0.51425 (18)	0.36573 (16)	0.0165 (9)	
C18B	0.6045 (6)	0.39529 (17)	0.45050 (15)	0.0202 (10)	
H18B	0.723951	0.389250	0.445666	0.024*	
C19B	0.5101 (5)	0.35823 (18)	0.48761 (16)	0.0184 (10)	
C20B	0.5908 (6)	0.31021 (18)	0.51064 (17)	0.0260 (11)	
H20B	0.706140	0.301647	0.501444	0.031*	
C21B	0.5073 (6)	0.27496 (19)	0.54638 (17)	0.0280 (11)	
H21B	0.564045	0.242095	0.561214	0.034*	
C22B	0.3384 (6)	0.28772 (19)	0.56077 (17)	0.0242 (11)	
C23B	0.2560 (6)	0.33560 (19)	0.53859 (16)	0.0230 (11)	
H23B	0.141060	0.344389	0.548127	0.028*	
C24B	0.3418 (6)	0.37046 (18)	0.50253 (16)	0.0223 (10)	
H24B	0.285049	0.403319	0.487676	0.027*	
C25B	0.0980 (7)	0.2648 (2)	0.61572 (18)	0.0385 (13)	
H25D	0.062675	0.235745	0.642073	0.058*	
H25E	0.017244	0.264353	0.586010	0.058*	
H25F	0.098807	0.303656	0.631938	0.058*	

### Atomic displacement parameters (Å^2^)

**Table:**

	*U*^11^	*U*^22^	*U*^33^	*U*^12^	*U*^13^	*U*^23^
O1A	0.0168 (16)	0.0271 (17)	0.0170 (14)	−0.0021 (14)	0.0002 (13)	0.0029 (13)
O2A	0.0096 (16)	0.0289 (17)	0.0359 (18)	0.0005 (14)	−0.0110 (14)	−0.0115 (14)
O3A	0.0121 (17)	0.0238 (16)	0.0239 (16)	0.0015 (14)	−0.0024 (13)	−0.0022 (13)
O4A	0.0165 (18)	0.0254 (17)	0.0350 (17)	0.0030 (15)	−0.0039 (14)	−0.0112 (14)
N1A	0.015 (2)	0.023 (2)	0.0234 (19)	0.0005 (17)	0.0000 (17)	−0.0075 (16)
N2A	0.009 (2)	0.022 (2)	0.0224 (19)	−0.0055 (17)	−0.0001 (17)	−0.0055 (16)
N3A	0.012 (2)	0.0173 (19)	0.0206 (18)	−0.0009 (16)	0.0018 (16)	−0.0023 (16)
C1A	0.028 (3)	0.013 (2)	0.018 (2)	0.002 (2)	0.002 (2)	−0.0031 (18)
C2A	0.024 (3)	0.026 (2)	0.020 (2)	0.002 (2)	0.001 (2)	−0.0015 (19)
C3A	0.035 (3)	0.027 (3)	0.031 (3)	−0.001 (2)	0.009 (3)	0.004 (2)
C4A	0.055 (4)	0.026 (3)	0.025 (3)	0.007 (3)	0.008 (3)	0.006 (2)
C5A	0.040 (3)	0.024 (3)	0.024 (2)	0.011 (2)	−0.003 (2)	0.000 (2)
C6A	0.028 (3)	0.020 (2)	0.020 (2)	0.008 (2)	−0.002 (2)	−0.0007 (19)
C7A	0.023 (3)	0.036 (3)	0.031 (2)	0.005 (2)	−0.007 (2)	0.000 (2)
C8A	0.014 (2)	0.029 (3)	0.019 (2)	0.002 (2)	−0.0037 (19)	−0.004 (2)
C9A	0.011 (2)	0.019 (2)	0.019 (2)	−0.0040 (19)	−0.0030 (18)	−0.0045 (18)
C10A	0.015 (2)	0.021 (2)	0.027 (2)	0.001 (2)	−0.004 (2)	−0.007 (2)
C11A	0.019 (3)	0.019 (2)	0.031 (2)	0.000 (2)	−0.005 (2)	0.000 (2)
C12A	0.023 (3)	0.027 (3)	0.020 (2)	−0.008 (2)	−0.008 (2)	0.005 (2)
C13A	0.015 (2)	0.028 (3)	0.017 (2)	−0.003 (2)	0.0006 (19)	−0.0027 (19)
C14A	0.009 (2)	0.020 (2)	0.016 (2)	−0.0055 (18)	−0.0037 (17)	−0.0030 (18)
C15A	0.012 (2)	0.021 (2)	0.017 (2)	−0.0018 (19)	0.0031 (19)	−0.0011 (18)
C16A	0.013 (3)	0.046 (3)	0.050 (3)	0.007 (2)	−0.015 (2)	−0.016 (3)
C17A	0.016 (3)	0.020 (2)	0.015 (2)	−0.003 (2)	0.0001 (18)	0.0039 (18)
C18A	0.011 (2)	0.023 (2)	0.022 (2)	−0.005 (2)	−0.0076 (19)	0.0033 (19)
C19A	0.012 (2)	0.018 (2)	0.021 (2)	0.0007 (19)	0.0021 (19)	0.0004 (18)
C20A	0.016 (2)	0.020 (2)	0.023 (2)	0.000 (2)	0.0005 (19)	−0.0037 (19)
C21A	0.013 (2)	0.024 (2)	0.025 (2)	0.005 (2)	−0.005 (2)	−0.001 (2)
C22A	0.019 (3)	0.021 (2)	0.022 (2)	−0.005 (2)	−0.001 (2)	−0.0031 (19)
C23A	0.014 (2)	0.022 (2)	0.031 (2)	0.003 (2)	−0.002 (2)	−0.002 (2)
C24A	0.014 (2)	0.024 (2)	0.027 (2)	−0.001 (2)	0.002 (2)	−0.004 (2)
C25A	0.029 (3)	0.023 (2)	0.030 (3)	0.001 (2)	0.002 (2)	−0.011 (2)
O1B	0.0268 (19)	0.045 (2)	0.0206 (16)	−0.0086 (17)	0.0018 (15)	−0.0079 (15)
O2B	0.0186 (18)	0.0185 (15)	0.0313 (17)	0.0018 (14)	0.0127 (14)	0.0030 (14)
O3B	0.0126 (17)	0.0274 (17)	0.0289 (16)	−0.0025 (14)	0.0033 (14)	0.0072 (14)
O4B	0.029 (2)	0.0335 (19)	0.0328 (18)	−0.0047 (16)	0.0034 (15)	0.0116 (16)
N1B	0.010 (2)	0.0217 (19)	0.0199 (18)	−0.0021 (17)	0.0042 (15)	0.0038 (16)
N2B	0.005 (2)	0.022 (2)	0.0218 (19)	−0.0023 (17)	0.0002 (16)	0.0020 (16)
N3B	0.011 (2)	0.0199 (19)	0.0192 (18)	−0.0034 (16)	−0.0014 (16)	−0.0002 (16)
C1B	0.035 (3)	0.025 (2)	0.019 (2)	−0.001 (2)	−0.006 (2)	−0.005 (2)
C2B	0.035 (3)	0.031 (3)	0.025 (2)	0.003 (2)	−0.006 (2)	−0.001 (2)
C3B	0.060 (4)	0.029 (3)	0.032 (3)	−0.002 (3)	−0.017 (3)	0.002 (2)
C4B	0.076 (5)	0.027 (3)	0.023 (3)	0.000 (3)	−0.010 (3)	−0.007 (2)
C5B	0.069 (4)	0.031 (3)	0.024 (3)	0.005 (3)	0.011 (3)	0.000 (2)
C6B	0.038 (3)	0.027 (3)	0.022 (2)	0.005 (2)	0.002 (2)	−0.001 (2)
C7B	0.042 (3)	0.051 (3)	0.038 (3)	0.004 (3)	0.008 (3)	−0.001 (3)
C8B	0.015 (2)	0.031 (3)	0.020 (2)	−0.004 (2)	0.0042 (19)	0.001 (2)
C9B	0.011 (2)	0.025 (2)	0.020 (2)	0.004 (2)	0.0000 (19)	0.0018 (19)
C10B	0.009 (2)	0.027 (2)	0.033 (3)	0.001 (2)	−0.002 (2)	0.007 (2)
C11B	0.016 (3)	0.017 (2)	0.042 (3)	0.004 (2)	0.004 (2)	−0.004 (2)
C12B	0.018 (3)	0.026 (3)	0.034 (3)	−0.003 (2)	0.005 (2)	−0.010 (2)
C13B	0.009 (2)	0.024 (2)	0.021 (2)	0.0005 (19)	0.0016 (19)	0.0013 (19)
C14B	0.009 (2)	0.017 (2)	0.019 (2)	−0.0006 (18)	0.0047 (18)	0.0006 (18)
C15B	0.010 (2)	0.019 (2)	0.015 (2)	−0.0022 (19)	−0.0055 (18)	0.0005 (18)
C16B	0.017 (3)	0.028 (2)	0.035 (3)	0.008 (2)	0.013 (2)	0.004 (2)
C17B	0.012 (2)	0.020 (2)	0.018 (2)	0.000 (2)	−0.0027 (18)	−0.0045 (18)
C18B	0.016 (2)	0.021 (2)	0.024 (2)	−0.003 (2)	−0.002 (2)	−0.0008 (19)
C19B	0.013 (2)	0.018 (2)	0.024 (2)	−0.0013 (19)	−0.0003 (19)	−0.0006 (19)
C20B	0.021 (3)	0.027 (2)	0.030 (3)	0.004 (2)	−0.001 (2)	−0.001 (2)
C21B	0.027 (3)	0.025 (3)	0.032 (3)	0.006 (2)	−0.002 (2)	0.007 (2)
C22B	0.025 (3)	0.023 (3)	0.024 (2)	−0.007 (2)	0.000 (2)	0.005 (2)
C23B	0.013 (3)	0.031 (3)	0.025 (2)	−0.003 (2)	0.001 (2)	0.004 (2)
C24B	0.021 (3)	0.021 (2)	0.026 (2)	−0.002 (2)	−0.004 (2)	0.004 (2)
C25B	0.035 (3)	0.042 (3)	0.038 (3)	−0.001 (3)	0.014 (3)	0.012 (2)

### Geometric parameters (Å, º)

**Table:**

O1A—C1A	1.383 (5)	O1B—C1B	1.378 (5)
O1A—C8A	1.443 (5)	O1B—C8B	1.427 (5)
O2A—N1A	1.390 (4)	O2B—N1B	1.395 (4)
O2A—C16A	1.443 (5)	O2B—C16B	1.438 (5)
O3A—C17A	1.226 (5)	O3B—C17B	1.231 (5)
O4A—C22A	1.360 (5)	O4B—C22B	1.361 (5)
O4A—C25A	1.431 (5)	O4B—C25B	1.439 (6)
N1A—C15A	1.276 (5)	N1B—C15B	1.274 (5)
N2A—C17A	1.347 (5)	N2B—C17B	1.345 (5)
N2A—N3A	1.395 (4)	N2B—N3B	1.377 (5)
N2A—H2NA	0.90 (5)	N2B—H2NB	0.87 (5)
N3A—C18A	1.279 (5)	N3B—C18B	1.278 (5)
C1A—C2A	1.391 (6)	C1B—C2B	1.381 (7)
C1A—C6A	1.401 (6)	C1B—C6B	1.406 (6)
C2A—C3A	1.387 (6)	C2B—C3B	1.377 (6)
C2A—H2AA	0.9500	C2B—H2BA	0.9500
C3A—C4A	1.384 (7)	C3B—C4B	1.380 (7)
C3A—H3AA	0.9500	C3B—H3BA	0.9500
C4A—C5A	1.377 (7)	C4B—C5B	1.377 (7)
C4A—H4AA	0.9500	C4B—H4BA	0.9500
C5A—C6A	1.383 (6)	C5B—C6B	1.390 (6)
C5A—H5AA	0.9500	C5B—H5BA	0.9500
C6A—C7A	1.509 (6)	C6B—C7B	1.495 (7)
C7A—H7AA	0.9800	C7B—H7BA	0.9800
C7A—H7AB	0.9800	C7B—H7BB	0.9800
C7A—H7AC	0.9800	C7B—H7BC	0.9800
C8A—C9A	1.499 (5)	C8B—C9B	1.504 (5)
C8A—H8AA	0.9900	C8B—H8BA	0.9900
C8A—H8AB	0.9900	C8B—H8BB	0.9900
C9A—C10A	1.396 (5)	C9B—C10B	1.389 (6)
C9A—C14A	1.400 (5)	C9B—C14B	1.393 (5)
C10A—C11A	1.381 (6)	C10B—C11B	1.375 (6)
C10A—H10A	0.9500	C10B—H10B	0.9500
C11A—C12A	1.376 (6)	C11B—C12B	1.380 (6)
C11A—H11A	0.9500	C11B—H11B	0.9500
C12A—C13A	1.388 (6)	C12B—C13B	1.385 (6)
C12A—H12A	0.9500	C12B—H12B	0.9500
C13A—C14A	1.397 (5)	C13B—C14B	1.387 (5)
C13A—H13A	0.9500	C13B—H13B	0.9500
C14A—C15A	1.493 (5)	C14B—C15B	1.499 (5)
C15A—C17A	1.499 (6)	C15B—C17B	1.506 (6)
C16A—H16A	0.9800	C16B—H16D	0.9800
C16A—H16B	0.9800	C16B—H16E	0.9800
C16A—H16C	0.9800	C16B—H16F	0.9800
C18A—C19A	1.455 (5)	C18B—C19B	1.463 (5)
C18A—H18A	0.9500	C18B—H18B	0.9500
C19A—C24A	1.393 (6)	C19B—C24B	1.395 (6)
C19A—C20A	1.394 (6)	C19B—C20B	1.394 (6)
C20A—C21A	1.378 (5)	C20B—C21B	1.375 (6)
C20A—H20A	0.9500	C20B—H20B	0.9500
C21A—C22A	1.391 (5)	C21B—C22B	1.398 (6)
C21A—H21A	0.9500	C21B—H21B	0.9500
C22A—C23A	1.391 (6)	C22B—C23B	1.389 (6)
C23A—C24A	1.377 (5)	C23B—C24B	1.384 (6)
C23A—H23A	0.9500	C23B—H23B	0.9500
C24A—H24A	0.9500	C24B—H24B	0.9500
C25A—H25A	0.9800	C25B—H25D	0.9800
C25A—H25B	0.9800	C25B—H25E	0.9800
C25A—H25C	0.9800	C25B—H25F	0.9800
			
C1A—O1A—C8A	116.9 (3)	C1B—O1B—C8B	117.0 (3)
N1A—O2A—C16A	107.5 (3)	N1B—O2B—C16B	107.3 (3)
C22A—O4A—C25A	117.5 (3)	C22B—O4B—C25B	117.4 (4)
C15A—N1A—O2A	112.2 (3)	C15B—N1B—O2B	111.7 (3)
C17A—N2A—N3A	120.3 (4)	C17B—N2B—N3B	119.1 (4)
C17A—N2A—H2NA	120 (3)	C17B—N2B—H2NB	122 (3)
N3A—N2A—H2NA	115 (3)	N3B—N2B—H2NB	118 (3)
C18A—N3A—N2A	113.2 (3)	C18B—N3B—N2B	115.8 (3)
O1A—C1A—C2A	124.2 (4)	O1B—C1B—C2B	124.2 (4)
O1A—C1A—C6A	114.9 (4)	O1B—C1B—C6B	113.9 (4)
C2A—C1A—C6A	120.9 (4)	C2B—C1B—C6B	121.9 (4)
C3A—C2A—C1A	119.7 (4)	C3B—C2B—C1B	119.6 (5)
C3A—C2A—H2AA	120.2	C3B—C2B—H2BA	120.2
C1A—C2A—H2AA	120.2	C1B—C2B—H2BA	120.2
C4A—C3A—C2A	119.8 (5)	C2B—C3B—C4B	119.9 (5)
C4A—C3A—H3AA	120.1	C2B—C3B—H3BA	120.1
C2A—C3A—H3AA	120.1	C4B—C3B—H3BA	120.1
C5A—C4A—C3A	120.0 (4)	C5B—C4B—C3B	120.2 (5)
C5A—C4A—H4AA	120.0	C5B—C4B—H4BA	119.9
C3A—C4A—H4AA	120.0	C3B—C4B—H4BA	119.9
C4A—C5A—C6A	121.6 (5)	C4B—C5B—C6B	121.8 (5)
C4A—C5A—H5AA	119.2	C4B—C5B—H5BA	119.1
C6A—C5A—H5AA	119.2	C6B—C5B—H5BA	119.1
C5A—C6A—C1A	118.0 (4)	C5B—C6B—C1B	116.6 (5)
C5A—C6A—C7A	122.1 (4)	C5B—C6B—C7B	122.3 (5)
C1A—C6A—C7A	119.9 (4)	C1B—C6B—C7B	121.2 (4)
C6A—C7A—H7AA	109.5	C6B—C7B—H7BA	109.5
C6A—C7A—H7AB	109.5	C6B—C7B—H7BB	109.5
H7AA—C7A—H7AB	109.5	H7BA—C7B—H7BB	109.5
C6A—C7A—H7AC	109.5	C6B—C7B—H7BC	109.5
H7AA—C7A—H7AC	109.5	H7BA—C7B—H7BC	109.5
H7AB—C7A—H7AC	109.5	H7BB—C7B—H7BC	109.5
O1A—C8A—C9A	108.9 (3)	O1B—C8B—C9B	107.8 (3)
O1A—C8A—H8AA	109.9	O1B—C8B—H8BA	110.2
C9A—C8A—H8AA	109.9	C9B—C8B—H8BA	110.2
O1A—C8A—H8AB	109.9	O1B—C8B—H8BB	110.2
C9A—C8A—H8AB	109.9	C9B—C8B—H8BB	110.2
H8AA—C8A—H8AB	108.3	H8BA—C8B—H8BB	108.5
C10A—C9A—C14A	118.5 (4)	C10B—C9B—C14B	118.4 (4)
C10A—C9A—C8A	120.3 (4)	C10B—C9B—C8B	119.2 (4)
C14A—C9A—C8A	121.2 (3)	C14B—C9B—C8B	122.4 (4)
C11A—C10A—C9A	121.0 (4)	C11B—C10B—C9B	121.3 (4)
C11A—C10A—H10A	119.5	C11B—C10B—H10B	119.3
C9A—C10A—H10A	119.5	C9B—C10B—H10B	119.3
C12A—C11A—C10A	120.3 (4)	C10B—C11B—C12B	120.0 (4)
C12A—C11A—H11A	119.9	C10B—C11B—H11B	120.0
C10A—C11A—H11A	119.9	C12B—C11B—H11B	120.0
C11A—C12A—C13A	120.0 (4)	C11B—C12B—C13B	119.7 (4)
C11A—C12A—H12A	120.0	C11B—C12B—H12B	120.1
C13A—C12A—H12A	120.0	C13B—C12B—H12B	120.1
C12A—C13A—C14A	120.1 (4)	C12B—C13B—C14B	120.2 (4)
C12A—C13A—H13A	120.0	C12B—C13B—H13B	119.9
C14A—C13A—H13A	120.0	C14B—C13B—H13B	119.9
C13A—C14A—C9A	120.1 (4)	C13B—C14B—C9B	120.3 (4)
C13A—C14A—C15A	118.4 (4)	C13B—C14B—C15B	118.6 (4)
C9A—C14A—C15A	121.5 (3)	C9B—C14B—C15B	121.1 (4)
N1A—C15A—C14A	126.4 (4)	N1B—C15B—C14B	129.1 (4)
N1A—C15A—C17A	115.0 (4)	N1B—C15B—C17B	114.0 (4)
C14A—C15A—C17A	118.6 (4)	C14B—C15B—C17B	117.0 (4)
O2A—C16A—H16A	109.5	O2B—C16B—H16D	109.5
O2A—C16A—H16B	109.5	O2B—C16B—H16E	109.5
H16A—C16A—H16B	109.5	H16D—C16B—H16E	109.5
O2A—C16A—H16C	109.5	O2B—C16B—H16F	109.5
H16A—C16A—H16C	109.5	H16D—C16B—H16F	109.5
H16B—C16A—H16C	109.5	H16E—C16B—H16F	109.5
O3A—C17A—N2A	124.9 (4)	O3B—C17B—N2B	124.4 (4)
O3A—C17A—C15A	121.6 (4)	O3B—C17B—C15B	120.7 (4)
N2A—C17A—C15A	113.5 (4)	N2B—C17B—C15B	114.8 (4)
N3A—C18A—C19A	123.5 (4)	N3B—C18B—C19B	121.1 (4)
N3A—C18A—H18A	118.2	N3B—C18B—H18B	119.5
C19A—C18A—H18A	118.2	C19B—C18B—H18B	119.5
C24A—C19A—C20A	118.5 (4)	C24B—C19B—C20B	118.1 (4)
C24A—C19A—C18A	118.6 (4)	C24B—C19B—C18B	122.1 (4)
C20A—C19A—C18A	122.8 (4)	C20B—C19B—C18B	119.8 (4)
C21A—C20A—C19A	120.5 (4)	C21B—C20B—C19B	121.5 (4)
C21A—C20A—H20A	119.7	C21B—C20B—H20B	119.2
C19A—C20A—H20A	119.7	C19B—C20B—H20B	119.2
C20A—C21A—C22A	120.1 (4)	C20B—C21B—C22B	119.7 (4)
C20A—C21A—H21A	119.9	C20B—C21B—H21B	120.2
C22A—C21A—H21A	119.9	C22B—C21B—H21B	120.2
O4A—C22A—C21A	115.3 (4)	O4B—C22B—C23B	124.7 (4)
O4A—C22A—C23A	124.7 (4)	O4B—C22B—C21B	115.5 (4)
C21A—C22A—C23A	120.0 (4)	C23B—C22B—C21B	119.8 (4)
C24A—C23A—C22A	119.3 (4)	C24B—C23B—C22B	119.8 (4)
C24A—C23A—H23A	120.4	C24B—C23B—H23B	120.1
C22A—C23A—H23A	120.4	C22B—C23B—H23B	120.1
C23A—C24A—C19A	121.5 (4)	C23B—C24B—C19B	121.2 (4)
C23A—C24A—H24A	119.3	C23B—C24B—H24B	119.4
C19A—C24A—H24A	119.3	C19B—C24B—H24B	119.4
O4A—C25A—H25A	109.5	O4B—C25B—H25D	109.5
O4A—C25A—H25B	109.5	O4B—C25B—H25E	109.5
H25A—C25A—H25B	109.5	H25D—C25B—H25E	109.5
O4A—C25A—H25C	109.5	O4B—C25B—H25F	109.5
H25A—C25A—H25C	109.5	H25D—C25B—H25F	109.5
H25B—C25A—H25C	109.5	H25E—C25B—H25F	109.5
			
C16A—O2A—N1A—C15A	−179.4 (3)	C16B—O2B—N1B—C15B	178.3 (3)
C17A—N2A—N3A—C18A	173.2 (3)	C17B—N2B—N3B—C18B	−178.8 (4)
C8A—O1A—C1A—C2A	2.0 (5)	C8B—O1B—C1B—C2B	3.2 (6)
C8A—O1A—C1A—C6A	−176.9 (3)	C8B—O1B—C1B—C6B	−176.4 (4)
O1A—C1A—C2A—C3A	−177.7 (4)	O1B—C1B—C2B—C3B	178.9 (4)
C6A—C1A—C2A—C3A	1.2 (6)	C6B—C1B—C2B—C3B	−1.6 (7)
C1A—C2A—C3A—C4A	−0.6 (6)	C1B—C2B—C3B—C4B	0.1 (7)
C2A—C3A—C4A—C5A	0.1 (7)	C2B—C3B—C4B—C5B	0.9 (7)
C3A—C4A—C5A—C6A	−0.2 (7)	C3B—C4B—C5B—C6B	−0.3 (8)
C4A—C5A—C6A—C1A	0.7 (6)	C4B—C5B—C6B—C1B	−1.1 (7)
C4A—C5A—C6A—C7A	179.4 (4)	C4B—C5B—C6B—C7B	178.7 (5)
O1A—C1A—C6A—C5A	177.7 (3)	O1B—C1B—C6B—C5B	−178.3 (4)
C2A—C1A—C6A—C5A	−1.2 (6)	C2B—C1B—C6B—C5B	2.1 (7)
O1A—C1A—C6A—C7A	−1.0 (6)	O1B—C1B—C6B—C7B	1.9 (6)
C2A—C1A—C6A—C7A	−179.9 (4)	C2B—C1B—C6B—C7B	−177.7 (4)
C1A—O1A—C8A—C9A	174.5 (3)	C1B—O1B—C8B—C9B	−174.6 (3)
O1A—C8A—C9A—C10A	−99.7 (4)	O1B—C8B—C9B—C10B	89.5 (5)
O1A—C8A—C9A—C14A	82.3 (4)	O1B—C8B—C9B—C14B	−91.0 (5)
C14A—C9A—C10A—C11A	0.7 (6)	C14B—C9B—C10B—C11B	−0.4 (6)
C8A—C9A—C10A—C11A	−177.3 (4)	C8B—C9B—C10B—C11B	179.2 (4)
C9A—C10A—C11A—C12A	−1.1 (6)	C9B—C10B—C11B—C12B	−0.1 (7)
C10A—C11A—C12A—C13A	0.2 (6)	C10B—C11B—C12B—C13B	0.1 (7)
C11A—C12A—C13A—C14A	1.0 (6)	C11B—C12B—C13B—C14B	0.3 (6)
C12A—C13A—C14A—C9A	−1.3 (6)	C12B—C13B—C14B—C9B	−0.7 (6)
C12A—C13A—C14A—C15A	175.8 (4)	C12B—C13B—C14B—C15B	−178.4 (4)
C10A—C9A—C14A—C13A	0.4 (6)	C10B—C9B—C14B—C13B	0.8 (6)
C8A—C9A—C14A—C13A	178.5 (4)	C8B—C9B—C14B—C13B	−178.8 (4)
C10A—C9A—C14A—C15A	−176.6 (4)	C10B—C9B—C14B—C15B	178.4 (4)
C8A—C9A—C14A—C15A	1.5 (6)	C8B—C9B—C14B—C15B	−1.2 (6)
O2A—N1A—C15A—C14A	0.7 (6)	O2B—N1B—C15B—C14B	0.2 (6)
O2A—N1A—C15A—C17A	179.3 (3)	O2B—N1B—C15B—C17B	179.9 (3)
C13A—C14A—C15A—N1A	90.4 (5)	C13B—C14B—C15B—N1B	−95.4 (5)
C9A—C14A—C15A—N1A	−92.5 (5)	C9B—C14B—C15B—N1B	87.0 (5)
C13A—C14A—C15A—C17A	−88.2 (5)	C13B—C14B—C15B—C17B	84.9 (5)
C9A—C14A—C15A—C17A	88.9 (5)	C9B—C14B—C15B—C17B	−92.7 (5)
N3A—N2A—C17A—O3A	7.3 (6)	N3B—N2B—C17B—O3B	−4.2 (6)
N3A—N2A—C17A—C15A	−174.4 (3)	N3B—N2B—C17B—C15B	175.2 (3)
N1A—C15A—C17A—O3A	147.3 (4)	N1B—C15B—C17B—O3B	−141.2 (4)
C14A—C15A—C17A—O3A	−33.9 (6)	C14B—C15B—C17B—O3B	38.6 (6)
N1A—C15A—C17A—N2A	−31.1 (5)	N1B—C15B—C17B—N2B	39.4 (5)
C14A—C15A—C17A—N2A	147.7 (4)	C14B—C15B—C17B—N2B	−140.9 (4)
N2A—N3A—C18A—C19A	−173.6 (3)	N2B—N3B—C18B—C19B	175.8 (3)
N3A—C18A—C19A—C24A	−169.1 (4)	N3B—C18B—C19B—C24B	−10.0 (6)
N3A—C18A—C19A—C20A	15.1 (6)	N3B—C18B—C19B—C20B	172.3 (4)
C24A—C19A—C20A—C21A	0.7 (6)	C24B—C19B—C20B—C21B	1.2 (6)
C18A—C19A—C20A—C21A	176.5 (4)	C18B—C19B—C20B—C21B	178.9 (4)
C19A—C20A—C21A—C22A	0.1 (6)	C19B—C20B—C21B—C22B	−1.0 (7)
C25A—O4A—C22A—C21A	179.5 (4)	C25B—O4B—C22B—C23B	−4.1 (6)
C25A—O4A—C22A—C23A	0.1 (6)	C25B—O4B—C22B—C21B	175.6 (4)
C20A—C21A—C22A—O4A	−179.8 (4)	C20B—C21B—C22B—O4B	−179.2 (4)
C20A—C21A—C22A—C23A	−0.5 (6)	C20B—C21B—C22B—C23B	0.5 (6)
O4A—C22A—C23A—C24A	179.4 (4)	O4B—C22B—C23B—C24B	179.4 (4)
C21A—C22A—C23A—C24A	0.1 (6)	C21B—C22B—C23B—C24B	−0.2 (6)
C22A—C23A—C24A—C19A	0.7 (6)	C22B—C23B—C24B—C19B	0.4 (6)
C20A—C19A—C24A—C23A	−1.1 (6)	C20B—C19B—C24B—C23B	−0.9 (6)
C18A—C19A—C24A—C23A	−177.1 (4)	C18B—C19B—C24B—C23B	−178.5 (4)

### Hydrogen-bond geometry (Å, º)

**Table:**

*D*—H···*A*	*D*—H	H···*A*	*D*···*A*	*D*—H···*A*
N2*A*—H2*NA*···O3*B*	0.90 (5)	2.04 (5)	2.797 (5)	141 (4)
N2*A*—H2*NA*···N3*B*	0.90 (5)	2.54 (5)	3.331 (5)	147 (4)
C16*A*—H16*B*···O3*A*^i^	0.98	2.51	3.353 (6)	144
C18*A*—H18*A*···N3*B*	0.95	2.60	3.494 (5)	158
N2*B*—H2*NB*···O3*A*^i^	0.87 (5)	2.50 (5)	2.995 (5)	117 (4)
N2*B*—H2*NB*···N3*A*^i^	0.87 (5)	2.29 (5)	3.155 (5)	172 (4)

Symmetry code: (i) *x*+1, *y*, *z*.
